# Brain but not serum BDNF levels are associated with structural alterations in the hippocampal regions in patients with drug-resistant mesial temporal lobe epilepsy

**DOI:** 10.3389/fnins.2023.1217702

**Published:** 2023-07-19

**Authors:** Elena A. Filimonova, Anton A. Pashkov, Galina I. Moysak, Anastasia Y. Tropynina, Svetlana Y. Zhanaeva, Anna A. Shvaikovskaya, Anna A. Akopyan, Konstantin V. Danilenko, Lyubomir I. Aftanas, Maria A. Tikhonova, Jamil A. Rzaev

**Affiliations:** ^1^FSBI "Federal Center of Neurosurgery", Novosibirsk, Russia; ^2^Department of Neurosurgery, Novosibirsk State Medical University, Novosibirsk, Russia; ^3^Biomedical School, South Ural State University, Chelyabinsk, Russia; ^4^Department of Neuroscience, Institute of Medicine and Psychology, Novosibirsk State University, Novosibirsk, Russia; ^5^Scientific Research Institute of Neurosciences and Medicine, Novosibirsk, Russia

**Keywords:** mesial temporal lobe epilepsy, drug-resistant epilepsy, BDNF, MR morphometry, FreeSurfer, hippocampus

## Abstract

Mesial temporal lobe epilepsy is the most common type of focal epilepsy, imposing a significant burden on the health care system worldwide. Approximately one-third of patients with this disease who do not adequately respond to pharmacotherapy are considered drug-resistant subjects. Despite having some clues of how such epileptic activity and resistance to therapy emerge, coming mainly from preclinical models, we still witness a scarcity of human data. To narrow this gap, in this study, we aimed to estimate the relationship between hippocampal and serum levels of brain-derived neurotrophic factor (BDNF), one of the main and most widely studied neurotrophins, and hippocampal subfield volumes in patients with drug-resistant mesial temporal epilepsy undergoing neurosurgical treatment. We found that hippocampal (but not serum) BDNF levels were negatively correlated with the contralateral volumes of the CA1 and CA4 subfields, presubiculum, subiculum, dentate gyrus, and molecular layer of the hippocampus. Taken together, these findings are generally in accordance with existing data, arguing for a proepileptic nature of BDNF effects in the hippocampus and related brain structures.

## Introduction

Epilepsy is a serious chronic nervous system disease characterized by recurrent seizures that strongly affect patients’ motor, cognitive and emotional functioning. With an annual incidence rate estimated to be approximately 68 per 100,000 persons (Fiest et al., 2017) and global costs of treating patients with epilepsy surpassing 100 billion dollars per year worldwide (Begley et al., 2022), epilepsy remains one of the leading causes of human disability, significant drop in quality of life, and mortality (Beghi, 2016; Saadi et al., 2016). Therefore, epilepsy has been an area of active research in medicine for decades. Despite apparent progress in finding new molecular and neuroimaging biomarkers and the development of novel approaches to treatment, approximately 30% of patients do not appropriately respond to the pharmacological therapy being conducted (Kalilani et al., 2018). These cases of drug-resistant epilepsy are frequently considered for further neurosurgical treatment aimed at resection of the epileptogenic zone found during neuroimaging and scalp or invasive EEG recordings. Generally, neurosurgical treatment is quite successful in eliminating or at least reducing the number and intensity of seizures, as exemplified by a large body of recent evidence showing that 53 to 84% of such patients are totally seizure-free after surgery at a follow-up point of as long as 1 year (Spencer and Huh, 2008; Vakharia et al., 2018). However, the health care community is still in an urgent need of objective biomarkers able to effectively and reliably predict the outcome of surgery.

Mesial temporal lobe epilepsy (MTLE) is the most common type of focal epilepsy in the clinical population (Zhou et al., 2020). MTLE is an umbrella term for focal epilepsies caused by pathological processes within any paramedian temporal structures, including the hippocampal formation, amygdala, entorhinal and parahippocampal gyri (Vinti et al., 2021). One of the most common histopathological diagnoses among adults with drug-resistant focal epilepsy requiring surgery is hippocampal sclerosis (HS; Blumcke et al., 2017). HS is histologically described as a loss of pyramidal cells in the CA1, CA3, or CA4 regions, whereas CA2 pyramidal and dentate gyrus granule cells are seizure resistant (Blümcke et al., 2012). The loss of nerve cells is associated with neural tissue fibrosis. Despite extensive research over the past several decades, the pathophysiology of HS is still not fully understood (Takano and Coulter, 2012). It has commonly been assumed that different aberrant mechanisms, such as ion channel disturbances (Muñoz et al., 2007; Sen et al., 2007), synaptic neurotransmission abnormalities (Mathern et al., 1999; Ben-Ari and Holmes, 2005), apoptosis (Yu et al., 2021), neuroinflammation (Gales and Prayson, 2017), and brain–blood barrier dysfunction (Van Vliet et al., 2007), are involved in this process. Furthermore, alterations in the expression of different neuropeptides have also been shown to participate in the development of HS (Thom, 2014). Another type of pathological pattern in TLE is gliosis without neuronal loss, called “no hippocampal sclerosis, gliosis only” or simply “gliosis only” (Blümcke et al., 2013). Reactive astrocytes are common in HS and other epileptic foci, and it is hypothesized that they increase tissue excitability and the generation of seizures. It is also not clear whether gliosis precedes a loss of neurons that leads to HS or constitutes a different disease (Wetherington et al., 2008).

Magnetic resonance imaging (MRI) is one of the most useful diagnostic techniques, which enables an effective identification of epilepsy substrates (Linera, 2019). It is routinely used in the initial assessment of patients with epilepsy, and the diagnosis of HS depends on positive signs on MRI. In drug-resistant cases, MRI is crucial in the selection of surgical candidates and contributes to the treatment plan. It is known that the use of high magnetic field scanners (3 T and more) with high-resolution sequences (both 3D and 2D) allows the detection of even very small lesions by increasing the tissue contrast and the signal-to-noise ratio (Sone, 2021).

In addition, the development of advanced MRI sequences as well as postprocessing techniques provides valuable additional information regarding the structural, physiological and metabolic depiction of brain tissue (Tsougos et al., 2019). One of the most perspective image postprocessing tools in the field of MTLE research is automatic MR-morphometry with FreeSurfer,^1^ which allows delineating the hippocampal formation with subsequent segmentation on anatomically different subfields (Iglesias et al., 2015). This technique is widely used (Sämann et al., 2022), and several recently published papers demonstrated that it could be helpful in the evaluation of patients with HS, both independently (Sone et al., 2016; Long et al., 2018; Costa et al., 2019) and as part of multimodal analysis (Rutland et al., 2018).

The structural alterations visible on MR images represent accumulated damage caused by disease progression. A disturbance of the exquisite balance between excitatory and inhibitory neural processes may lead to the emergence of epileptogenic zones wherein epileptic activity is generated. Neuroplasticity plays a fundamental role in forming new synapses and pruning ineffective synapses, regulating the excitation to inhibition ratio (E:I ratio), and thus is of special interest to researchers investigating the cellular and molecular mechanisms of epilepsy. Among all members of the large family of neurotrophins that mediate neuroplasticity-associated tissue reorganization, brain-derived neurotrophic factor (BDNF) holds a unique place. BDNF is well established to be involved in the pathogenesis of a multitude of neurological and psychiatric disorders, such as Parkinson’s (Palasz et al., 2020) and Alzheimer’s diseases (Ng et al., 2019), epilepsy (Iughetti et al., 2018), depression (Yang et al., 2020), anxiety disorders (Suliman et al., 2013) and many others. Dysfunction of synaptic plasticity at the molecular level could be a common denominator for all of the abovementioned pathologies. The results of early studies on BDNF involvement in the emergence and spread of epileptic activity in the brain were ambiguous, with some researchers arguing for the antiepileptic nature of BDNF and others taking the opposite side (Koyama and Ikegaya, 2005; Simonato et al., 2006). Over time, the accumulated evidence has tipped the scales toward the stance shared by proponents of the proepileptic nature of hippocampal neurotrophic factors (Scharfman et al., 2005; Iughetti et al., 2018; Porcher et al., 2018).

It should be mentioned, however, that the overwhelming majority of studies are preclinical studies conducted on experimental animals in laboratory settings, whereas human data are scarce. Rare clinical trials performed in small-sized samples of patients with epilepsy undergoing surgical treatment are of great value and worth noting here. For instance, Martinez-Levy et al. reported that the expression of BDNF exon VI was increased in the hippocampus of patients with MTLE compared to the corresponding levels of hippocampal BDNF in individuals who died of causes other than psychiatric or neurological diseases (Martínez-Levy et al., 2016, 2018). In another early paper, in agreement with other studies, the authors gauged the BDNF mRNA level in the hippocampi of temporal lobe epilepsy patients and found that it was increased in a group of patients with hippocampal sclerosis (Mathern et al., 1997). Even more remarkable results were presented by Takahashi et al. (1999). They not only observed an elevated hippocampal level of BDNF in MTLE patients but also demonstrated that the same did not hold for other neurotrophins (nerve growth factor and neurotrophin 3) investigated in that study. Along the same lines, a study by Murray et al. adds to the growing list of studies evidencing the positive association between BDNF levels and the epileptogenic capacity of hippocampal neural circuits (Murray et al., 2000).

In most cases, when patients are not referred to neurosurgical treatment, one of the readily available options for clinicians to investigate, although indirectly, the role of BDNF in epileptogenesis is to measure its level in the patient’s serum. The number of such studies using BDNF serum levels in MTLE patients vastly outnumber those directly measuring BDNF in hippocampal tissue, which we have mentioned earlier. The results of these studies consistently reveal markedly decreased serum BDNF levels in patients with MTLE (Chen et al., 2016, 2017; Panina et al., 2023). However, noteworthy is a couple of published studies that did not segregate patients with focal epilepsy into subtypes and found no significant differences in serum BDNF levels between control and epilepsy groups (Hong et al., 2014; Nowroozi et al., 2021).

In this study, we set out to assess the relationship between BDNF measured in hippocampal samples and serum taken from epileptic patients during surgery and morphometric parameters of hippocampal subfields.

## Materials and methods

### Patients

Subjects in this study were patients diagnosed with drug-resistant MTLE according to the diagnostic guidelines (Kwan et al., 2010) who were surgically treated at our hospital from 2021 to 2022. Twenty patients (13 males and 7 females, 19–47 years of age) with morphologically proven hippocampal sclerosis or gliosis participated in the study. All patients underwent noninvasive video-EEG monitoring and high-resolution brain MRI before surgery. In addition, some patients had previously undergone implantation of electrodes for deep brain stimulation (patient #7), electrodes for invasive EEG recording (patients #4, 14, 18, 20) or cortical epileptogenic zone resection surgery (patient #15). All patients underwent anterior temporal lobectomy. Detailed information about the patients is provided in Table 1; Supplementary Table 1. Each patient signed a written informed consent form to participate in the study. The study was conducted in accordance with the Declaration of Helsinki and approved by the local Ethics Committee of the Federal Center for Neurosurgery, Novosibirsk, Russia (protocol No. 1 dated 1 March 2019).

**Table 1:** Patient’s clinical information.

ID	Age	Gender	Side	Seizure onset age	Duration of the disease (years)	Precipitating factors	AED before surgery	Previous surgical history	Histology	Engel
1	36	M	R	26	10	N/A	Valproic acid, lacosamide, zonisamide	No	HS	I
2	29	F	L	1	28	DPT vaccine	Lacosamide, lamotrigine, oxcarbazepine, levetiracetam	Stereotactic biopsy of the left hemispheric lesion (FCD ILAE type II)	HG	IV
3	22	M	L	6	16	N/A	Carbamazepine, levetiracetam	No	HG	N/A
4	35	M	R	26	9	N/A	Levetiracetam, oxcarbazepine	Invasive EEG with electrodes placement in both temporal areas	HS	IV
5	30	F	L	14	16	N/A	Lacosamide	No	HS	N/A
6	30	F	L	26	4	N/A	Topiramate, levetiracetam	No	HG	I
7	38	M	L	5	33	N/A	Benzobarbital, carbamazepine	DBS of anterior thalamus for 3 years (no effect)	HS	I
8	19	M	L	3	16	N/A	Lacosamide, perampanel	No	HG	I
9	39	M	L	4	35	N/A	Topiramate, oxcarbazepine	No	HG	I
10	35	F	R	15	20	N/A	Lacosamide, topiramate	No	HS	IV
11	31	M	L	18	13	N/A	Sodium valproate, lacosamide	No	HS	I
12	39	M	R	18	21	N/A	Sodium valproate, perampanel	No	HS	N/A
13	37	F	R	9	28	Tick-borne encephalitis	Levetiracetam, lamotrigine	No	HS	IV
14	31	F	L	12	19	N/A	Carbamazepine, levetiracetam, clonazepam	Invasive EEG-monitoring with intra-axial left temporal lobe electrodes placement	HS	I
15	39	M	R	20	19	Cortical epileptogenic zone (ipsilateral temporal lobe)	Levetiracetam, lamotrigine, lacosamide	Cortical epileptogenic zone resection (partial effect)	HS	IV
16	40	F	R	8	32	Meningitis	Benzobarbital, carbamazepine, levetiracetam	No	HS	I
17	39	M	L	11	28	Tick-borne encephalitis	Sodium valproate, lacosamide	No	HS	IV
18	47	M	L	41	6	Alcohol-induced	Sodium valproate, lamotrigine	Invasive EEG-monitoring with intra-axial bilateral temporal electrodes placement	HS	I
19	47	M	L	2.5	44.5	N/A	Valproic acid, levetiracetam, lamotrigine	No	HS	IV
20	29	M	L	24	5	Multiple developmental anomalies	Carbamazepine, levetiracetam	Invasive EEG-monitoring with intra-axial bilateral temporal electrodes placement	HS	I

L, left side; R, right side; M, male; F, female; AED, anti-epileptic drugs; DBS, deep brain stimulation; HS, hippocampal sclerosis; HG, hippocampal gliosis; N/A, information is not available.

### BDNF in serum

Peripheral blood samples for BDNF analysis were taken before surgery after overnight fasting. Blood was collected in anticoagulant-free tubes with the clotting activator and kept for 1 h at a temperature of approximately 21°C–23°C. After centrifugation (2,000 × g for 10 min) and aliquoting, the samples were frozen at −20°C. Serum BDNF levels (in pg./mL) were measured by multiplex solid-phase analysis using a multiplex analyzer of proteins and nucleic acids (MILLIPLEX Luminex 200, Merck KGaA, Germany) and xMAP technology using Multiplex Assays reagent for BDNF (HNDG3MAG-36 K-01 Human Neurodegenerative Disease Magnetic Bead Panel 3, MERK).

### Immunohistochemical staining

**Figure 1:**
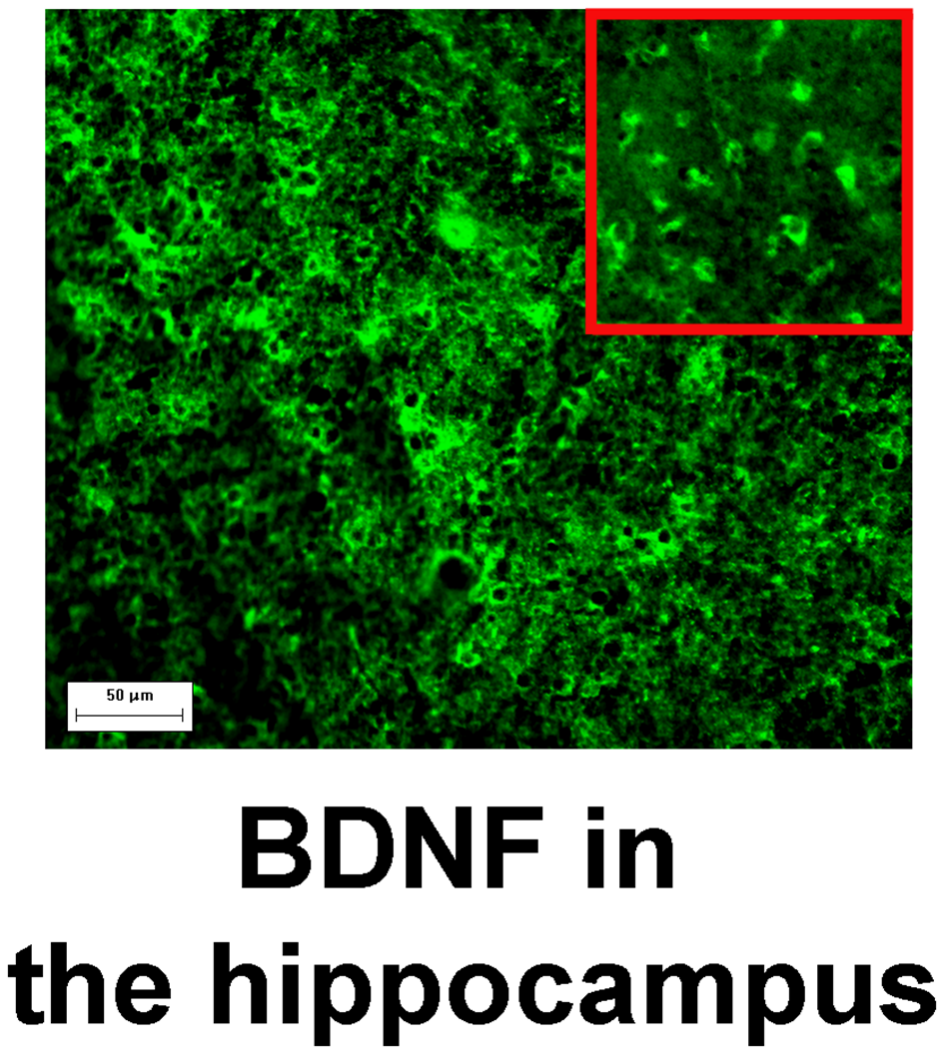
BDNF immunoreactivity in the hippocampus. Magnification, 200×; scale bar, 50 μm. High zoom image of BDNF-positive cells is shown in the inset.

BDNF levels in the hippocampal tissue were measured using immunohistochemical analysis. All morphological samples (approximately 0.5 cm^3^ in volume) from MTLE patients were stored immediately after resection in fresh 4% paraformaldehyde in PBS for 24 h and then postfixed in PBS containing 30% sucrose at 4°C. After being immersed in the embedding Tissue-Tek O.C.T. compound (Sakura Finetek, United States), the samples were frozen and stored at −70°C until sectioning into 30-μm-thick slices with a cryostat HistoSafe MicroCut—SADV (China). Slices were incubated for 1 h in 5% BSA with 0.1% Triton X-100 in PBS, followed by overnight incubation at 4°C with primary antibodies against BDNF (1200, NB100-98682, Novus Biologicals, United States). After incubation with the respective secondary antibodies diluted 1:600 (goat-anti-rabbit Alexa Flur 488, ab150077, Abcam, United Kingdom), the slices were covered with Fluoromount containing 4*′*,6-diamidino-2-phenylindole (DAPI; Abcam). The fluorescence images were finally obtained by an Axioplan 2 (Carl Zeiss) imaging microscope and then analyzed in Image-Pro Plus Software 6.0 (Media Cybernetics, MD, United States). Figure 1 shows an example of BDNF immunoreactivity in the hippocampus. Fluorescence intensity associated with the expression of BDNF was measured as background-corrected optical density with subtraction of staining signals of the nonimmunoreactive regions in the images converted to grayscale. Negative control samples with the omitted primary antibody emitted only a minimal autofluorescent signal. Four tissue slices per patient and five areas per slice were analyzed, and the mean fluorescence intensity was measured. For each image acquisition, all imaging parameters were kept the same.

### MRI data acquisition

MR imaging data were acquired using a 3 T system (Ingenia, Philips Healthcare, The Netherlands) equipped with a 16-channel receiver head coil. Brain magnetic resonance imaging was performed according to the HARNESS protocol (Federico et al., 2020) and included high-resolution 3D T1-WI, T2-WI, FLAIR, and SWI sequences, as well as 2D T2-WI with high in-plane resolution with oblique coronal orientation of slices perpendicular to the long axis of the hippocampi. The total acquisition time was approximately 45 min. The T1-WI high-resolution sequence (3D TFE in the sagittal plane) had the following parameters: TR—6.56 ms, TE—2.95 ms, FOV—256*256 mm, flip angle—8, matrix—256*256, and slice thickness—1 mm.

### Automatic MR morphometry

Fully automatic surface-based MR morphometry of high-resolution T1-WI was performed with FreeSurfer v7.2.0 software (see footnote 1) and included basic and subcortical segmentation, with subsequent segmentation of hippocampal subfields (Iglesias et al., 2015). The results were visually checked by the neuroradiologist for each subject. Parameters such as volumes of hippocampi were used for the following analysis after basic and subcortical segmentation with “recon-all” script. Volumes of structures such as CA1, CA2/3, CA4, dentate gyrus, molecular layer, subiculum, and presubiculum were used for further analysis after hippocampal subfield segmentation (with “segmentHA_T1.sh” script). In all cases, segmentation results were visually checked by a neuroradiologist. An example of MRI data processing is shown in Supplementary Figure 1.

### Statistical analysis

Descriptive statistics were presented in the form of median (interquartile range, IQR), given the small sample size and nonnormal distribution of the data. The relationship between measured variables was assessed with the Spearman correlation coefficient and FDR correction for multiple comparisons. To increase the robustness of inferences made on the small-sized sample, we additionally ran a Bayesian correlation analysis, where a Bayesian factor (BF) exceeding 3 is generally taken as some evidence favoring one model over another (Dienes, 2014). The Chi-squared test was used for categorical data analysis. The Mann–Whitney test was utilized for comparison of metric variables grouped by one categorical variable, such as gender or side of pathology. A *p*-value of 0.05 was considered a threshold for evaluating statistically significant associations. We provide all the calculated values, complemented with 95% confidence intervals, in Supplementary Table 2. All statistical analyses were run in R (v. 4.2.2, 2022).

## Results

### Demographic data and clinical information

A total of 20 patients (13 males, 7 females) with drug-resistant MTLE were included in the study. The median age of the recruited patients was 35.5 (IQR = 9). The duration of the disease had a median value of 19 with IQR = 15.75. Male and female patients did not differ in age (U = 33.5, *p* = 0.36) or duration of the disease (U = 50.5, *p* = 0.72). Seven patients had right-sided disease-affected hippocampi, and 13 patients presented with left-sided hippocampi. Similarly, patients with right- and left-sided pathology did not differ in the same variables (U = 28, *p* = 0.18 for age; U = 42.5, *p* = 0.84 for disease duration).

Detailed information about presurgical EEG and MRI data for each patient is provided in Supplementary Table 1. The pathomorphological analysis revealed 15 cases of hippocampal sclerosis and five cases of hippocampal gliosis (Table 1). Surgery outcomes were defined according to Engel’s classification (Engel et al., 2003) 6 months after the anterior temporal lobectomy, and the results are shown in Table 1.

### Serum BDNF level and BDNF level in hippocampal formation

Median concentrations of BDNF in the patient’s brain and serum were 56.96 a.u. (IQR = 43.32) and 3488.75 pg./mL (IQR = 1132.74), respectively. Hippocampal and serum BDNF levels did not differ between the male and female groups (U = 48, *p* = 0.88 and U = 42.5, *p* = 0.75, respectively). Similarly, patients with either side of pathology did not differ from each other in the levels of BDNF (U = 56, *p* = 0.44 for hippocampal BDNF level; U = 27, *p* = 0.32 for serum BDNF level). Statistically significant relations between BDNF levels and duration of the disease were not detected (R = 0.4, *p* = 0.08 for hippocampal BDNF; r = 0.4, *p* = 0.16 for serum BDNF). In the same way, age was not correlated with BDNF levels (r = −0.12, *p* = 0.62 and r = −0.05, *p* = 0.84, respectively). A significant relationship between BDNF protein expression levels in the resected hippocampus and serum BDNF levels was also not found (R = 0.46, *p* = 0.48, FDR corrected; BF = 0.69).

### Serum BDNF level and results of hippocampal MR morphometry

There were no statistically significant associations between serum BDNF level and volume of the resected hippocampus, as well as the volumes of different hippocampal subfields according to MR-morphometry (*p* > 0.1 for all comparisons, FDR corrected, not shown). Similarly, correlations between serum BDNF level and volume of the contralateral hippocampal subfields were not found (*p* > 0.1 for all comparisons, FDR corrected; not shown). Exact *p* values for all measured variables under pairwise comparison can be found in Supplementary Table 2.

### BDNF level in the hippocampus and results of hippocampal MR-morphometry

We did not reveal any statistically significant associations between BDNF protein expression level within the resected (ipsilateral) hippocampus and the volume of the resected hippocampus, as well as the volumes of its different subfields according to MR-morphometry (*p* > 0.1 in all comparisons, FDR corrected, not shown).

**Figure 2:**
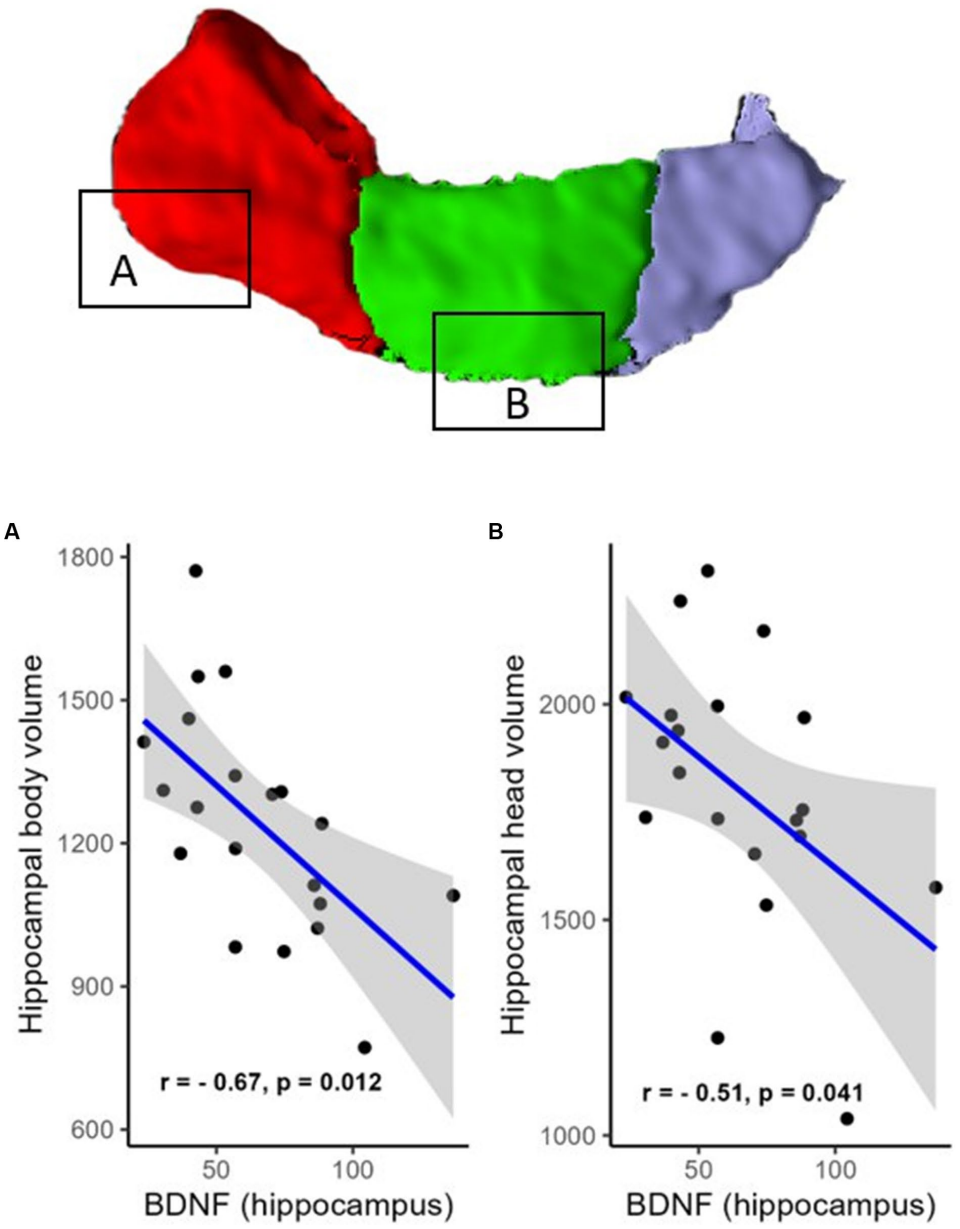
Relationship (Spearman’s correlation) between hippocampal BDNF level and the volumes of hippocampal regions (**A**—head and **B**—body). Placed at the top of the figure is a rendering of automatic hippocampal segmentation based on MR images with capital letters **(A,B)** referring to the corresponding plots below. The gray zone around the blue line represents a 95% confidence interval for the correlation coefficient. Units of measurement: volumes of hippocampal structures—mm^3^; hippocampal BDNF (optical density of BDNF immunofluorescence); a.u., arbitrary units.

**Figure 3:**
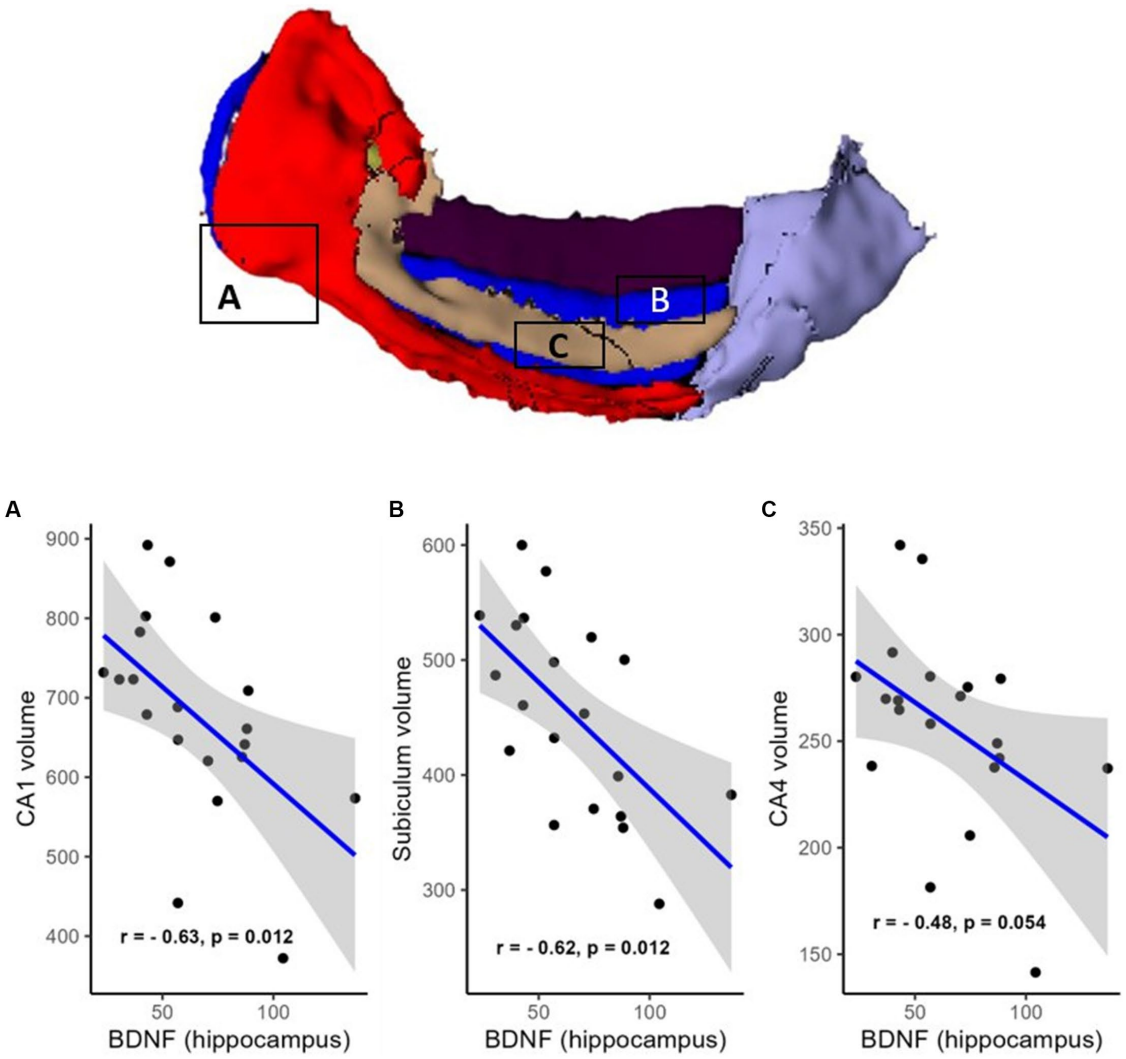
Relationship (Spearman’s correlation) between hippocampal BDNF level and the volumes of hippocampal subfields (**A**—CA1, **B**—CA4, and **C—**subiculum). Placed at the top of the figure is a rendering of automatic hippocampal segmentation based on MR images with capital letters **(A–C)** referring to the corresponding plots below. The gray zone around the blue line represents a 95% confidence interval for the correlation coefficient. Units of measurement: volumes of hippocampal structures—mm^3^; hippocampal BDNF (optical density of BDNF immunofluorescence); a.u., arbitrary units.

**Figure 4:**
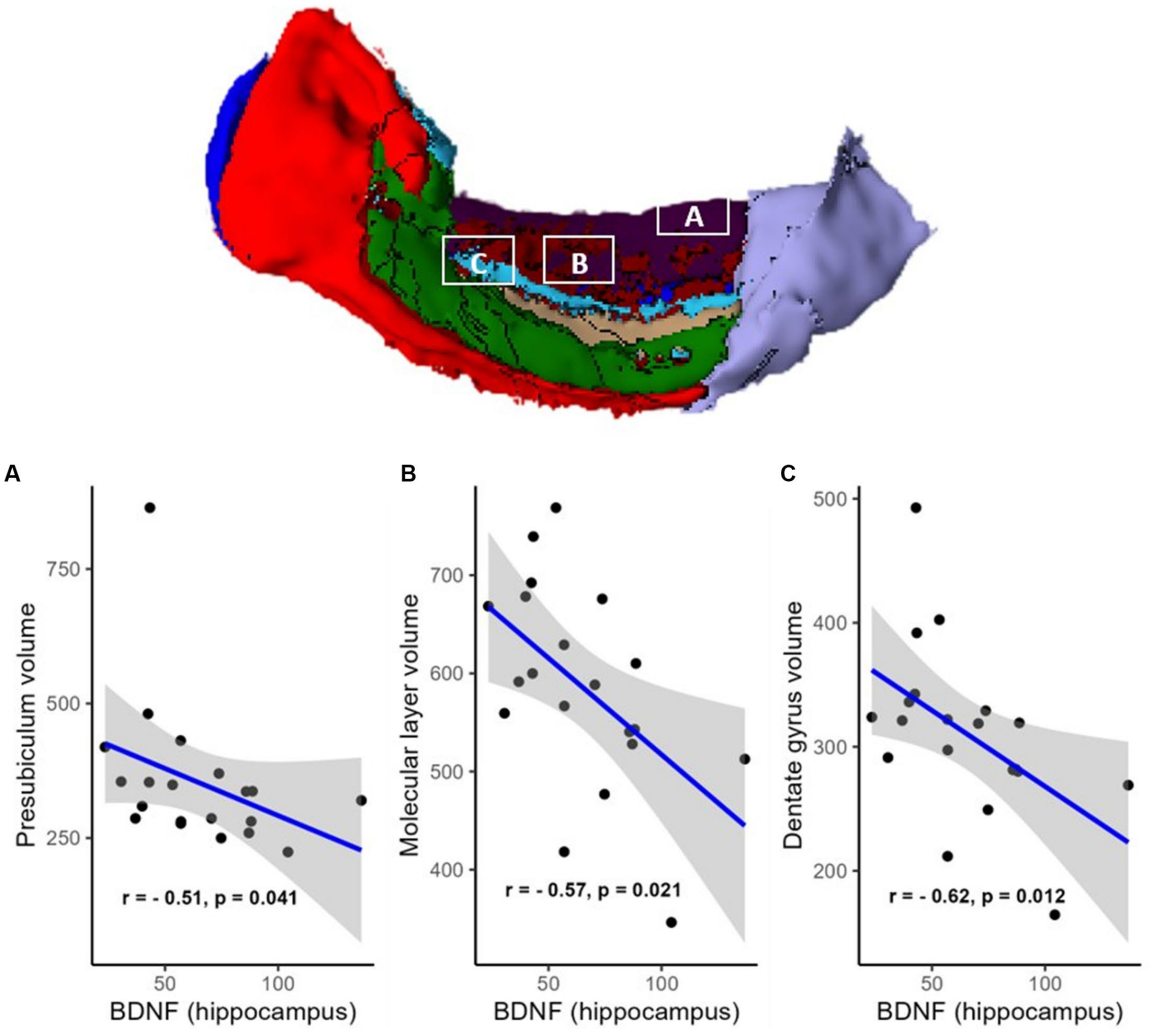
Relationship (Spearman’s correlation) between hippocampal BDNF level and the volumes of hippocampal subfields [**A**—presubiculum, **B**—molecular layer (shown in dark red), and **C**—dentate gyrus (shown in light blue)]. The area colored green is CA3, and the area colored light violet is the hippocampal tail (no statistically significant associations with BDNF). Placed at the top of the figure is a rendering of automatic hippocampal segmentation based on MR images with capital letters **(A–C)** referring to the corresponding plots below. The gray zone around the blue line represents a 95% confidence interval for the correlation coefficient. Units of measurement: volumes of hippocampal structures—mm^3^, hippocampal BDNF (optical density of BDNF immunofluorescence); a.u., arbitrary units.

However, a negative correlation between BDNF protein expression levels within the resected hippocampus and the volume of the contralateral hippocampus was found (R = −0.61, *p* = 0.012, FDR corrected; BF = 4.95; not shown). There were also negative correlations in areas such as the hippocampal head (R = −0.51, *p* = 0.041, FDR corrected; BF = 2.53; Figure 2B), hippocampal body (R = −0.67, *p* = 0.012, FDR corrected; BF = 11.47; Figure 2A), CA1 (R = −0.63, *p* = 0.012, FDR corrected; BF = 4.81; Figure 3A), subiculum (R = −0.62, *p* = 0.012, FDR corrected; BF = 12.07; Figure 3B), CA4 (R = −0.48, *p* = 0.054, FDR corrected; BF = 2.19; Figure 3C), presubiculum (R = −0.51, *p* = 0.041, FDR corrected; BF = 1.25; Figure 4A), dentate gyrus (R = −0.62, *p* = 0.012, FDR corrected; BF = 3.42; Figure 4B), and molecular layer (R = −0.57, *p* = 0.021, FDR corrected; BF = 4.67; Figure 4C). No statistically significant relationship was found between contralateral CA2/3 volume and hippocampal BDNF level (adjusted *p* > 0.1, not shown).

## Discussion

The search for biomarkers of disease progression, severity and resistance to therapy in patients with epilepsy is a pivotal direction in fundamental and applied medical science. In the current study, we focused on estimating the relationship between BDNF protein levels in the hippocampi or blood serum of patients with MTLE and the results of MR-based automatic segmentation of hippocampal subfields. Our results are generally in agreement with those published so far, showing that BDNF could indeed be an informative biomarker reflecting epilepsy-associated neural tissue reorganization. Specifically, we found that patients with more pronounced increases in hippocampal BDNF had smaller hippocampal volumes. In a similar vein, we discovered that different hippocampal subfields were not equally affected by the disease. Among the regions exhibiting the most statistically significant results were the CA1, subiculum, dentate gyrus and molecular layer. The CA1 and subiculum subfields have consistently been shown to exhibit a lowered threshold for the generation of epileptic activity and, therefore, are one of the first brain areas to undergo structural alterations detectable with high-resolution MRI (Stafstrom, 2005; Schoene-Bake et al., 2014; Kitaura et al., 2018; Santos et al., 2021). To our knowledge, this is the first attempt to compare serum and hippocampal BDNF levels with structural alterations in the hippocampal subfields according to MR morphometry.

We did not reveal any relationship between serum and hippocampal BDNF levels in this study, which is an interesting finding. Despite the fact that blood BDNF concentration reflects brain-tissue BDNF levels in some animals (Klein et al., 2011), the human data in this field are scarce. Notably, in humans, BDNF is widely expressed outside of the central nervous system, namely, in white blood cells, platelets, vascular endothelium, smooth muscles, and other peripheral tissues (Shvaikovskaya et al., 2020). Thus, its serum levels are likely to be influenced by peripheral sources rather than central BDNF levels. On the other hand, it is believed that serum BDNF levels are decreased in patients with MTLE compared to healthy controls (Chen et al., 2016, 2017), whereas hippocampal BDNF expression levels are increased (Murray et al., 2000). However, according to the results of the study by Iughetti and colleagues, serum BDNF can also be increased in human subjects suffering from epileptic seizures (Iughetti et al., 2018). It is therefore possible that hippocampal and serum BDNF concentrations change in different directions and with different dynamics, depending on the presence or absence of other confounding clinical variables. Thus, it seems likely that hippocampal and blood serum BDNF concentrations may be uncorrelated in patients with MTLE. We also did not find any correlations between serum BDNF levels and the results of MR morphometry of the hippocampal subfields. It is difficult to unambiguously ascertain the underlying causes of the absence of associations, but the results of a recently conducted preregistered study investigating a relationship between serum BDNF levels and hippocampal subfield volumes in healthy participants pointed in the same direction as our own. The authors did not find any significant correlations between BDNF and morphometric features of different hippocampal subfields and concluded that serum BDNF might have a limited predictive value for the morphological differences of the hippocampal structure (Puhlmann et al., 2021).

Surprisingly, we did not reveal any relationship between the levels of BDNF (both within the brain tissue and serum) and the volumes of the hippocampal subfields on the side of resection. The reason may be that all of our patients had severe drug-resistant epilepsy, a long history of disease, and obvious atrophy of the affected hippocampus. It is not clear from the available literature whether hippocampal volume loss is associated with the duration of MTLE (Kim et al., 2015; Caciagli et al., 2017; Kreilkamp et al., 2018; Wu et al., 2020). Loss of hippocampal volume was found to correlate with disease duration (Wu et al., 2020). Furthermore, Kim et al. showed an inverse relationship between disease duration and ipsilateral CA1 volume (Kim et al., 2015). Interestingly, this volume loss in CA1 contributes to the reduction in antiepileptic drug efficiency in models of chronic epilepsy. In contrast, despite also showing visible cell loss, the extent of neural degeneration unfolding in the CA4 region has been positively associated with the surgery outcome (King and Spencer, 1995; Savitr Sastri et al., 2014). One plausible explanation for this observation may be that the CA4 is strongly involved in the process of spreading epileptic activity to the contralateral hippocampus. Therefore, preventing this spreading, ipsilateral CA4 degeneration could protect the normally functioning hippocampus and, perhaps, make it possible for it to take over the functions of the affected hippocampus (King and Spencer, 1995). Meanwhile, Kreilkamp et al. reported that the volumes of hippocampal subfields were not correlated with clinical variables such as disease severity and disease duration (Kreilkamp et al., 2018). It has further been stated that HS can occur immediately after an initial precipitating injury; therefore, it may well be independent of chronicity and severity of the MTLE, since the majority of neurons might have already been malfunctioning or undergone irreversible changes by the time first seizures manifest (Pitkänen and Lukasiuk, 2011). Extending these findings, a series of papers have been published in the last decade, indicating a strong negative association between serum BDNF level and epilepsy duration (Hong et al., 2014; Chen et al., 2016). In contrast to the abovementioned statements, in our study, neither hippocampal nor blood serum BDNF correlated with disease duration. Notably, similar findings were reported in the recently published paper by Poniatowski et al. (2021).

We found a number of negative associations between hippocampal BDNF levels and volumes of different subfields of the contralateral hippocampus. We assume that it could reflect the spread of the disease, as contralateral hippocampus involvement in the MTLE process is well known and not uncommon (Pulsipher et al., 2007; Wu et al., 2020). Furthermore, a less favorable surgery outcome was demonstrated in patients with unilateral MTLE but the presence of contralateral hippocampus volume loss (Conz et al., 2014). In our study, negative correlations between BDNF levels and volumes of the contralateral CA1, subiculum, dentate gyrus and molecular layer subfields were revealed. There was also a trend toward a negative correlation between BDNF levels and the volume of the contralateral CA4 subfield. On the other hand, there were no correlations between CA2/3 volume and BDNF level. In general, these results are in line with the existing theoretical models of hippocampal epileptogenesis in MTLE. The CA1 and CA4 hippocampal subfields, which predominantly contain glutamatergic pyramidal neurons, are known to be the most susceptible to cell loss (Blümcke et al., 2013). However, CA2 pyramidal and dentate gyrus granule cells are relatively resistant to seizure damage with the involvement of these subfields only in late-stage disease (Thom, 2014). This can be explained by a direct excitotoxic effect of glutamate (Davolio and Greenamyre, 1995; Dillon et al., 2008) since CA2 is not part of the trisynaptic circuit (Takano and Coulter, 2012); additionally, a difference in damage susceptibility within the CA1 and CA3 subfields appears to be mediated by microglial reactivity (Lana et al., 2020). Recently, the subiculum was found to be a source of epileptogenic activity in patients with HS with its subsequent spreading to CA1, CA3, and CA4 areas (Kitaura et al., 2018); this may also be the reason for the observed volume reduction.

Another important part of epileptogenesis is neuroimmunity, which has been extensively studied during the last decade (Lima Giacobbo et al., 2019; Dai et al., 2021; Wang et al., 2021). Neuroinflammation is closely related to the development of epilepsy (Lima Giacobbo et al., 2019). For example, the protein of the high mobility group B1 (HMGB1) has shown elevated expression and an increased proportion of translocation from the nucleus to the cytoplasm in patients with epilepsy (Dai et al., 2021; Li et al., 2022). Therefore, HMGB1 and its signaling pathways may be a direction for antiepileptic drug therapy (Wang et al., 2021). However, BDNF and neuroinflammation signaling appear to be closely connected (Lima Giacobbo et al., 2019; Porter and O’Connor, 2022; Wang et al., 2022). For instance, BDNF has been shown to alleviate hippocampal neuroinflammation (Han et al., 2021). Therefore, another possible explanation for the increase in hippocampal BDNF expression in patients with MTLE is an attempt to compensate for the neuroinflammation burst.

Taken together, the results of this study provide additional evidence supporting the proepileptic action profile of BDNF and its predominantly negative relationship with hippocampal subfield volumes. However, after decades of investigation, given the scarcity of human data and conflicting findings, we still cannot confidently rely on BDNF as a biomarker for epilepsy severity, chronicity or treatment outcome prediction.

### Limitations

This study has several limitations. First, we had a small sample size (n = 20), preventing us from making any far-reaching conclusions except the preliminary ones. Second, the participants were heterogeneous: we included patients not only with idiopathic hippocampal sclerosis but also with different precipitating factors in our study. Furthermore, some patients in our study presented with histopathologically proven hippocampal “gliosis only.” Another possible limitation is that we recruited only patients with a long history of drug-resistant MTLE since these are notoriously the prime candidates for surgery. In addition, some of our patients had previously been surgically treated (one patient had DBS and another one had a resection of small temporal pole epileptogenic focus), which could affect the neuroplasticity processes involved in the progression of the disease and thus append some bias in our results. There are some limitations in our assessment of BDNF in hippocampal tissue. For example, our study did not perform an analysis of the distribution of BDNF in different cell types; analysis of the upstream and downstream factors of BDNF was also not included in our study and should be addressed in future research. Additionally, being limited by the sample size, we were not able to quantitatively estimate the effect of antiepileptic drugs taken by patients on underlying hippocampal structural modifications. Finally, most of the inferences made in this study relied on the results of correlation analysis. While undoubtedly useful and valuable, caution should be taken in regards to generalization of these findings due to our inability to make any causal statements about the role BDNF plays in epileptogenesis. Further studies on larger samples with the application of advanced statistical tools and intervention techniques are clearly needed.

## Conclusion

We found that hippocampal BDNF levels were negatively correlated with the volumes of the different subfields of the contralateral (but not ipsilateral) hippocampus in patients with drug-resistant MTLE. These findings are generally in accordance with existing data, arguing for a proepileptic nature of BDNF action.

## Data availability statement

The raw data supporting the conclusions of this article will be made available by the authors, without undue reservation.

## Ethics statement

The studies involving human participants were reviewed and approved by local Ethics Committee of the Federal Center for Neurosurgery, Novosibirsk, Russia (protocol No. 1 dated 1 March 2019). The patients/participants provided their written informed consent to participate in this study.

## Author contributions

LA, KD, GM, and JR: conceptualization. EF, AP, SZ, AA, and MT: methodology. EF and AP: formal analysis. GM and MT: data curation. EF, AP, AT, SZ, AS, and AA: investigation. GM: resources. EF, AP, and GM: writing—original draft preparation. MT, JR, and LA: writing—review and editing. AP: visualization. LA, KD, and JR: supervision. MT: project administration and funding acquisition. All authors contributed to the article and approved the submitted version.

## Funding

This study was funded by the Russian Science Foundation (grant no. 22-25-00588).

## Conflict of interest

The authors declare that the research was conducted in the absence of any commercial or financial relationships that could be construed as a potential conflict of interest.

## Publisher’s note

All claims expressed in this article are solely those of the authors and do not necessarily represent those of their affiliated organizations, or those of the publisher, the editors and the reviewers. Any product that may be evaluated in this article, or claim that may be made by its manufacturer, is not guaranteed or endorsed by the publisher.

## Footnotes

^1^ https://surfer.nmr.mgh.harvard.edu

## Abbreviations

AED: Antiepileptic drugs
a.u.: Arbitrary units
BDNF: Brain-derived neurotrophic factor
EEG: Electroencephalography
HS: Hippocampal sclerosis
ILAE: International League Against Epilepsy
MTLE: Mesial temporal lobe epilepsy
MRI: Magnetic resonance imaging
PBS: Phosphate-buffered saline
