# Supplementary figures and images for: Brain but not serum BDNF levels are associated with structural alterations in the hippocampal regions in patients with drug-resistant mesial temporal lobe epilepsy
Source: Front Neurosci. 2023 Jul 19;17:1217702. doi: 10.3389/fnins.2023.1217702 (PMC10395949; doi:10.3389/fnins.2023.1217702)

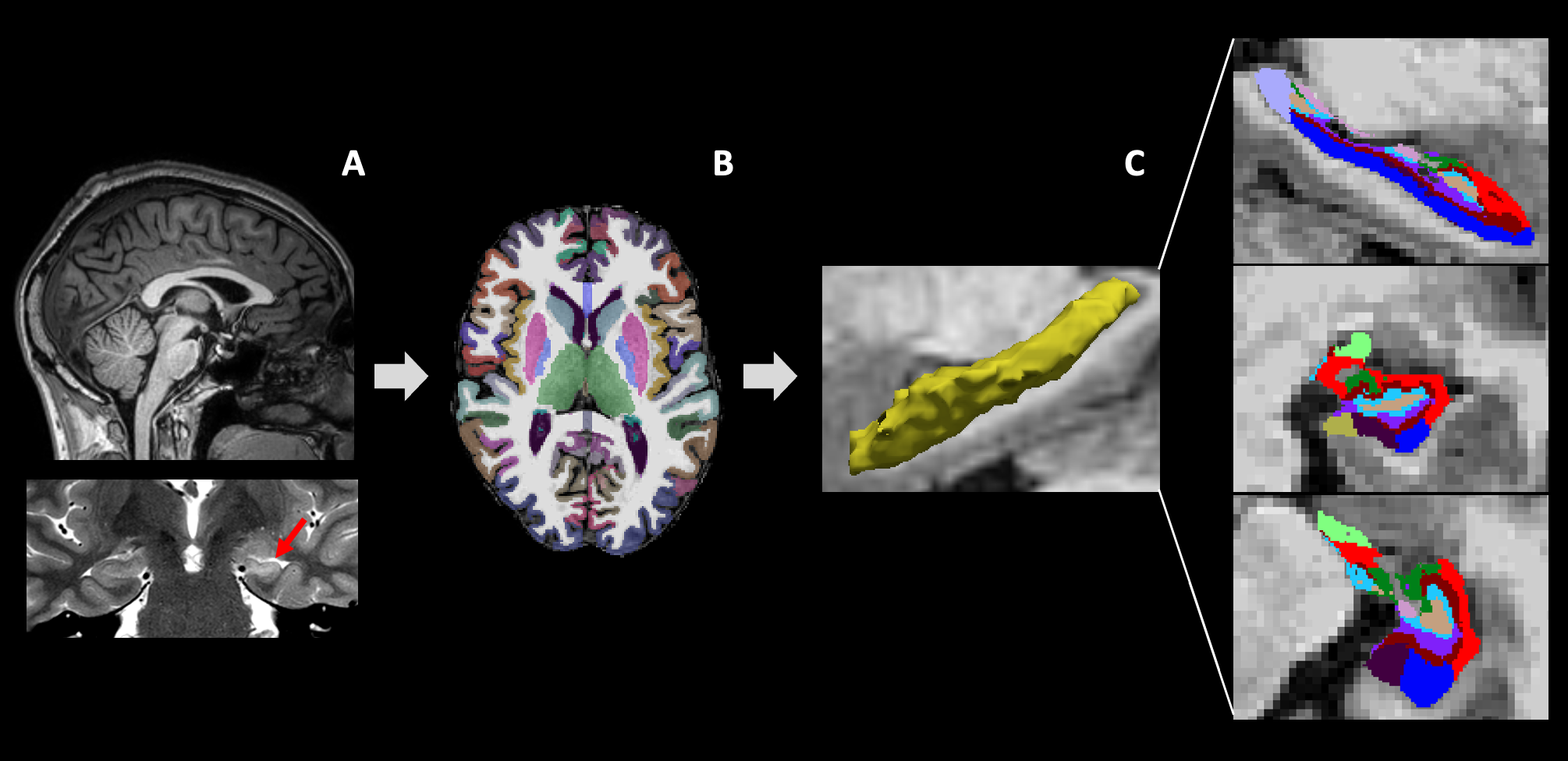

Supplement: Supplementary file 1 [file Image_1.png]
