# Supplementary material for: Brain but not serum BDNF levels are associated with structural alterations in the hippocampal regions in patients with drug-resistant mesial temporal lobe epilepsy
Source: Front Neurosci. 2023 Jul 19;17:1217702. doi: 10.3389/fnins.2023.1217702 (PMC10395949; doi:10.3389/fnins.2023.1217702)
Supplement: Supplementary file 2 [file Table_1.docx]

*Supplementary Table 1. Conventional MRI and scalp EEG data*

| **N** | **Age**  **(years)** | **Gender** | **Side** | **High-resolution MRI (3T)** | **Scalp EEG**  **(sources of epileptiform activity)** |
| --- | --- | --- | --- | --- | --- |
| 1 | 36 | m | R | Small right temporal pole encephaloceles; no structural changes in hippocampi | Right anterior-middle temporal area (F8, F10, T4, T10) |
| 2 | 29 | f | L | Extensive dysplastic changes in the left hemisphere with frontal lobe, insula and basal ganglia involvement | Left fronto-temporal area (F3-C3, F7-T3, F9-T9) |
| 3 | 22 | m | L | Left hippocampal sclerosis | Left anterior-middle temporal area (F7-T3, F9-T9) |
| 4 | 35 | m | R | Linear gliotic changes in left temporal lobe (history of invasive EEG-monitoring); no structural changes in hippocampi | Right temporal lobe (invasive) |
| 5 | 30 | f | L | Left hippocampal sclerosis | Left temporal lobe (F7-T3, F9-T9) |
| 6 | 30 | f | L | Left hippocampal sclerosis; left medial anterior temporal area small arachnoid cyst | Left anterior-middle temporal area (F7-F9-T3-T9) |
| 7 | 38 | m | L | Left hippocampal sclerosis | Left temporal lobe (F7-T3, F9-T9) |
| 8 | 19 | m | L | Left mesial temporal lobe cortical signal alteration (FCD ILAE type I); no hippocampal structural changes | Left temporal lobe (F7-T3, F9-T9) |
| 9 | 39 | m | L | Left hippocampal sclerosis | Left fronto-temporal area (F7, F9, T3, T9) |
| 10 | 35 | f | R | Right hippocampal sclerosis | Right temporal lobe (F8-T4, F10-T10, T4-T6, T10-P10) |
| 11 | 31 | m | L | Left hippocampal sclerosis | Left temporal lobe (F7-T3, F9-T9) |
| 12 | 39 | m | R | Right hippocampal sclerosis | Right anterior-middle temporal area (F8-T4, F10-T10) |
| 13 | 37 | f | R | Right temporal lobe atrophy and gliosis with hippocampus involvement (post-infection changes) | Right temporal lobe (F8-T4-T6, F10-T10-P10) |
| 14 | 31 | f | L | Left hippocampal sclerosis; left temporal lobe linear gliotic changes (history of invasive EEG-monitoring) | Left anterior-middle temporal area (F7-T3, F9-T9) |
| 15 | 39 | m | R | Right temporal lobe post-surgical changes (history of cortical epileptogenic zone resection), right hippocampal sclerosis | Right temporal lobe (F8-T4, F10-T10) |
| 16 | 40 | f | R | Right hippocampal sclerosis | Right temporal lobe (F8-T4, F10-T10) |
| 17 | 39 | m | L | No structural changes in temporal lobes | Left temporal lobe (F7-T3-T5, F9-T9-P9) |
| 18 | 47 | m | L | Linear gliotic changes in both temporal lobes (history of invasive EEG-monitoring), no other brain structural alterations | Right temporal lobe (F8-T4, F10-T10) |
| 19 | 47 | m | L | Left hippocampal sclerosis | Left temporal lobe (F7-T3, F9-T9) |
| 20 | 29 | m | L | Multiple developmental anomalies (bilateral PVH, hippocampal malrotation, right cerebellar hemisphere hypo- and dysplasia) | Left temporal lobe (F7-T3, F9-T9) + Invasive |

Abbreviations: FCD - focal cortical dysplasia, ILAE - International League Against Epilepsy, PVH - periventricular heterotopia
