# Supplementary material for: Brain but not serum BDNF levels are associated with structural alterations in the hippocampal regions in patients with drug-resistant mesial temporal lobe epilepsy
Source: Front Neurosci. 2023 Jul 19;17:1217702. doi: 10.3389/fnins.2023.1217702 (PMC10395949; doi:10.3389/fnins.2023.1217702)
Supplement: Supplementary file 3 [file Table_2.docx]

**Contralateral side**

BDNF_L is a level of BDNF measured in leukocytes (not described in the article due to

absence of any associations with other measures and structural alterations).

All p-values in these tables are FDR-corrected ones.

IOD - optic density

Correlations between hippocampal BDNF and other variables

**Parameter1 | Parameter2 | rho | 95% CI | S | p**

**--------------------------------------------------------------------------------**

**BDNF (IOD) | BDNF serum | 0.46 | [-0.03, 0.77] | 524.77 | 0.084**

**BDNF (IOD) | BDNF_L (IOD) | 0.24 | [-0.24, 0.63] | 1010.00 | 0.329**

**BDNF (IOD) | Hippocampal body | -0.67 | [-0.86, -0.31] | 2224.00 | 0.012***

**BDNF (IOD) | Hippocampal head | -0.51 | [-0.78, -0.07] | 2010.00 | 0.041***

**BDNF (IOD) | Hippocampal tail | -0.36 | [-0.70, 0.11] | 1808.00 | 0.150**

**BDNF (IOD) | Subiculum | -0.62 | [-0.84, -0.23] | 2156.00 | 0.012***

**BDNF (IOD) | CA1 | -0.63 | [-0.84, -0.24] | 2162.00 | 0.012***

**BDNF (IOD) | Presubiculum | -0.51 | [-0.78, -0.07] | 2008.00 | 0.041***

**BDNF (IOD) | Molecular layer | -0.57 | [-0.81, -0.16] | 2092.00 | 0.021***

**BDNF (IOD) | GC-ML-DG | -0.62 | [-0.84, -0.24] | 2160.00 | 0.012***

**BDNF (IOD) | CA2/3 | -0.33 | [-0.68, 0.15] | 1764.00 | 0.185**

**BDNF (IOD) | CA4 | -0.48 | [-0.77, -0.03] | 1968.00 | 0.054**

**BDNF (IOD) | Hippocampus (Vol mm3) | -0.61 | [-0.83, -0.22] | 2146.00 | 0.012***

**BDNF (IOD) | Duration of the disease | 0.40 | [-0.06, 0.72] | 796.19 | 0.108**

**BDNF (IOD) | Age | -0.12 | [-0.54, 0.35] | 1488.90 | 0.616**

Correlations between serum BDNF and other variables

**Parameter1 | Parameter2 | rho | 95% CI | S | p**

**------------------------------------------------------------------------------**

**BDNF serum | BDNF (IOD) | 0.46 | [-0.03, 0.77] | 524.77 | 0.418**

**BDNF serum | BDNF_L (IOD) | -0.04 | [-0.51, 0.45] | 1009.02 | 0.925**

**BDNF serum | Hippocampal body | -0.34 | [-0.70, 0.17] | 1296.17 | 0.552**

**BDNF serum | Hippocampal head | -0.17 | [-0.60, 0.34] | 1129.08 | 0.710**

**BDNF serum | Hippocampal tail | 0.02 | [-0.46, 0.50] | 945.99 | 0.925**

**BDNF serum | Subiculum | -0.30 | [-0.68, 0.20] | 1263.15 | 0.552**

**BDNF serum | CA1 | -0.31 | [-0.69, 0.20] | 1268.15 | 0.552**

**BDNF serum | Presubiculum | -0.16 | [-0.59, 0.34] | 1126.08 | 0.710**

**BDNF serum | Molecular layer | -0.17 | [-0.60, 0.33] | 1136.09 | 0.710**

**BDNF serum | GC-ML-DG | -0.09 | [-0.54, 0.41] | 1056.04 | 0.904**

**BDNF serum | CA2/3 | -0.22 | [-0.63, 0.29] | 1180.11 | 0.710**

**BDNF serum | CA4 | -0.20 | [-0.62, 0.30] | 1167.10 | 0.710**

**BDNF serum | Hippocampus (Vol mm3) | -0.31 | [-0.69, 0.19] | 1272.16 | 0.552**

**BDNF serum | Duration of the disease | 0.52 | [ 0.05, 0.80] | 466.40 | 0.411**

**BDNF serum | Age | -0.05 | [-0.52, 0.44] | 1017.35 | 0.925**

**Bayesian analysis**

Correlations between hippocampal BDNF and other variables

**Parameter1 | Parameter2 | rho | 95% CI | pd | % in ROPE | Prior | BF**

**--------------------------------------------------------------------------------------------------------------**

**BDNF (IOD) | BDNF serum | 0.17 | [-0.20, 0.58] | 80.35% | 27.27% | Beta (3 +- 3) | 0.689**

**BDNF (IOD) | BDNF_L (IOD) | 0.03 | [-0.36, 0.41] | 56.93% | 39.23% | Beta (3 +- 3) | 0.481**

**BDNF (IOD) | Hippocampal body | -0.49 | [-0.77, -0.12] | 99.62%** | 3.28% | Beta (3 +- 3) | 11.47****

**BDNF (IOD) | Hippocampal head | -0.37 | [-0.69, -0.03] | 96.95% | 8.25% | Beta (3 +- 3) | 2.53**

**BDNF (IOD) | Hippocampal tail | -0.30 | [-0.63, 0.10] | 92.12% | 14.17% | Beta (3 +- 3) | 1.44**

**BDNF (IOD) | Subiculum | -0.50 | [-0.77, -0.19] | 99.75%** | 1.27% | Beta (3 +- 3) | 12.07****

**BDNF (IOD) | CA1 | -0.41 | [-0.70, -0.07] | 97.95%* | 4.38% | Beta (3 +- 3) | 4.81***

**BDNF (IOD) | Presubiculum | -0.28 | [-0.61, 0.08] | 92.65% | 15.53% | Beta (3 +- 3) | 1.25**

**BDNF (IOD) | Molecular layer | -0.42 | [-0.70, -0.07] | 98.00%* | 4.45% | Beta (3 +- 3) | 4.67***

**BDNF (IOD) | GC-ML-DG | -0.39 | [-0.70, -0.02] | 97.60%* | 6.60% | Beta (3 +- 3) | 3.42***

**BDNF (IOD) | CA2/3 | -0.29 | [-0.60, 0.09] | 91.65% | 14.12% | Beta (3 +- 3) | 1.44**

**BDNF (IOD) | CA4 | -0.34 | [-0.65, 0.04] | 95.53% | 9.32% | Beta (3 +- 3) | 2.19**

**BDNF (IOD) | Hippocampus (Vol mm3) | -0.43 | [-0.71, -0.09] | 98.90%* | 3.48% | Beta (3 +- 3) | 4.95***

**BDNF (IOD) | Duration of the disease | 0.22 | [-0.16, 0.58] | 87.08% | 21.68% | Beta (3 +- 3) | 0.856**

**BDNF (IOD) | Age | -0.16 | [-0.51, 0.23] | 78.90% | 27.77% | Beta (3 +- 3) | 0.654**

Correlations between serum BDNF and other variables

**Parameter1 | Parameter2 | rho | 95% CI | pd | % in ROPE | Prior | BF**

**---------------------------------------------------------------------------------------------------------**

**BDNF serum | BDNF (IOD) | 0.16 | [-0.24, 0.53] | 77.75% | 27.32% | Beta (3 +- 3) | 0.689**

**BDNF serum | BDNF_L (IOD) | -0.10 | [-0.49, 0.30] | 67.03% | 32.17% | Beta (3 +- 3) | 0.558**

**BDNF serum | Hippocampal body | -0.20 | [-0.58, 0.19] | 83.55% | 22.45% | Beta (3 +- 3) | 0.792**

**BDNF serum | Hippocampal head | -0.12 | [-0.51, 0.30] | 72.45% | 30.30% | Beta (3 +- 3) | 0.591**

**BDNF serum | Hippocampal tail | -0.05 | [-0.42, 0.34] | 58.73% | 36.85% | Beta (3 +- 3) | 0.505**

**BDNF serum | Subiculum | -0.18 | [-0.56, 0.19] | 81.05% | 26.07% | Beta (3 +- 3) | 0.723**

**BDNF serum | CA1 | -0.18 | [-0.54, 0.20] | 80.75% | 24.88% | Beta (3 +- 3) | 0.716**

**BDNF serum | Presubiculum | -0.03 | [-0.42, 0.36] | 56.00% | 36.48% | Beta (3 +- 3) | 0.499**

**BDNF serum | Molecular layer | -0.15 | [-0.55, 0.24] | 75.42% | 27.95% | Beta (3 +- 3) | 0.644**

**BDNF serum | GC-ML-DG | 0.04 | [-0.36, 0.44] | 57.70% | 34.85% | Beta (3 +- 3) | 0.510**

**BDNF serum | CA2/3 | -0.23 | [-0.59, 0.16] | 86.40% | 19.50% | Beta (3 +- 3) | 0.930**

**BDNF serum | CA4 | -0.16 | [-0.54, 0.23] | 77.58% | 28.20% | Beta (3 +- 3) | 0.663**

**BDNF serum | Hippocampus (Vol mm3) | -0.17 | [-0.55, 0.21] | 79.70% | 26.92% | Beta (3 +- 3) | 0.710**

**BDNF serum | Duration of the disease | 0.33 | [-0.06, 0.66] | 94.23% | 10.25% | Beta (3 +- 3) | 1.97**

**BDNF serum | Age | -0.05 | [-0.45, 0.33] | 61.00% | 34.38% | Beta (3 +- 3) | 0.518**

**Ipsilateral side**

Correlations between hippocampal BDNF and other variables

**Parameter1 | Parameter2 | rho | 95% CI | S | p**

**------------------------------------------------------------------------------**

**BDNF (IOD) | BDNF serum | 0.46 | [-0.03, 0.77] | 524.77 | 0.477**

**BDNF (IOD) | BDNF_L (IOD) | 0.24 | [-0.24, 0.63] | 1010.00 | 0.477**

**BDNF (IOD) | Hippocampal body | -0.31 | [-0.67, 0.17] | 1738.00 | 0.477**

**BDNF (IOD) | Hippocampal head | -0.20 | [-0.60, 0.28] | 1596.00 | 0.486**

**BDNF (IOD) | Hippocampal tail | -0.25 | [-0.63, 0.23] | 1662.00 | 0.477**

**BDNF (IOD) | Subiculum | -0.24 | [-0.63, 0.24] | 1650.00 | 0.477**

**BDNF (IOD) | CA1 | -0.22 | [-0.61, 0.26] | 1624.00 | 0.486**

**BDNF (IOD) | Presubiculum | -0.27 | [-0.65, 0.21] | 1692.00 | 0.477**

**BDNF (IOD) | Molecular layer | -0.24 | [-0.63, 0.24] | 1650.00 | 0.477**

**BDNF (IOD) | GC-ML-DG | -0.17 | [-0.58, 0.31] | 1550.00 | 0.523**

**BDNF (IOD) | CA2/3 | -0.19 | [-0.59, 0.29] | 1586.00 | 0.486**

**BDNF (IOD) | CA4 | -0.26 | [-0.64, 0.22] | 1674.00 | 0.477**

**BDNF (IOD) | Hippocampus (Vol mm3) | -0.14 | [-0.57, 0.35] | 1300.00 | 0.567**

**BDNF (IOD) | Duration of the disease | 0.40 | [-0.06, 0.72] | 796.19 | 0.477**

Correlations between serum BDNF and other variables

**Parameter1 | Parameter2 | rho | 95% CI | S | p**

**------------------------------------------------------------------------------**

**BDNF serum | BDNF (IOD) | 0.46 | [-0.03, 0.77] | 524.77 | 0.390**

**BDNF serum | BDNF_L (IOD) | -0.04 | [-0.51, 0.45] | 1009.02 | 0.871**

**BDNF serum | Hippocampal body | -0.22 | [-0.63, 0.29] | 1181.11 | 0.871**

**BDNF serum | Hippocampal head | -0.14 | [-0.58, 0.36] | 1104.07 | 0.871**

**BDNF serum | Hippocampal tail | -0.10 | [-0.55, 0.40] | 1066.05 | 0.871**

**BDNF serum | Subiculum | -0.11 | [-0.56, 0.39] | 1073.05 | 0.871**

**BDNF serum | CA1 | -0.19 | [-0.62, 0.31] | 1157.10 | 0.871**

**BDNF serum | Presubiculum | -0.13 | [-0.57, 0.37] | 1097.07 | 0.871**

**BDNF serum | Molecular layer | -0.15 | [-0.58, 0.36] | 1112.07 | 0.871**

**BDNF serum | GC-ML-DG | 0.04 | [-0.45, 0.51] | 927.98 | 0.871**

**BDNF serum | CA2/3 | -0.16 | [-0.59, 0.35] | 1120.08 | 0.871**

**BDNF serum | CA4 | -0.20 | [-0.62, 0.31] | 1159.10 | 0.871**

**BDNF serum | Hippocampus (Vol mm3) | -0.08 | [-0.55, 0.43] | 879.04 | 0.871**

**BDNF serum | Duration of the disease | 0.52 | [ 0.05, 0.80] | 466.40 | 0.384**

**Bayesian analysis**

Correlations between hippocampal BDNF and other variables

**Parameter1 | Parameter2 | rho | 95% CI | pd | % in ROPE | Prior | BF**

**---------------------------------------------------------------------------------------------------------**

**BDNF (IOD) | BDNF serum | 0.17 | [-0.23, 0.52] | 78.35% | 26.82% | Beta (3 +- 3) | 0.689**

**BDNF (IOD) | BDNF_L (IOD) | 0.04 | [-0.35, 0.41] | 56.88% | 37.67% | Beta (3 +- 3) | 0.481**

**BDNF (IOD) | Hippocampal body | -0.29 | [-0.61, 0.09] | 92.80% | 14.50% | Beta (3 +- 3) | 1.29**

**BDNF (IOD) | Hippocampal head | -0.16 | [-0.52, 0.22] | 78.75% | 28.05% | Beta (3 +- 3) | 0.665**

**BDNF (IOD) | Hippocampal tail | -0.24 | [-0.56, 0.15] | 88.60% | 18.80% | Beta (3 +- 3) | 0.964**

**BDNF (IOD) | Subiculum | -0.20 | [-0.57, 0.18] | 84.55% | 22.43% | Beta (3 +- 3) | 0.822**

**BDNF (IOD) | CA1 | -0.19 | [-0.56, 0.19] | 82.58% | 24.73% | Beta (3 +- 3) | 0.768**

**BDNF (IOD) | Presubiculum | -0.16 | [-0.52, 0.22] | 77.25% | 27.77% | Beta (3 +- 3) | 0.655**

**BDNF (IOD) | Molecular layer | -0.21 | [-0.57, 0.16] | 84.47% | 22.73% | Beta (3 +- 3) | 0.878**

**BDNF (IOD) | GC-ML-DG | -0.20 | [-0.53, 0.23] | 84.72% | 23.05% | Beta (3 +- 3) | 0.787**

**BDNF (IOD) | CA2/3 | -0.16 | [-0.51, 0.23] | 80.12% | 26.82% | Beta (3 +- 3) | 0.667**

**BDNF (IOD) | CA4 | -0.25 | [-0.60, 0.12] | 89.58% | 17.88% | Beta (3 +- 3) | 1.07**

**BDNF (IOD) | Hippocampus (Vol mm3) | -0.17 | [-0.56, 0.21] | 79.88% | 26.77% | Beta (3 +- 3) | 0.684**

**BDNF (IOD) | Duration of the disease | 0.21 | [-0.15, 0.58] | 85.12% | 23.82% | Beta (3 +- 3) | 0.856**

Correlations between serum BDNF and other variables

**Parameter1 | Parameter2 | rho | 95% CI | pd | % in ROPE | Prior | BF**

**---------------------------------------------------------------------------------------------------------**

**BDNF serum | BDNF (IOD) | 0.17 | [-0.22, 0.53] | 79.60% | 26.47% | Beta (3 +- 3) | 0.689**

**BDNF serum | BDNF_L (IOD) | -0.10 | [-0.48, 0.28] | 68.70% | 32.07% | Beta (3 +- 3) | 0.558**

**BDNF serum | Hippocampal body | -0.19 | [-0.52, 0.24] | 79.92% | 24.88% | Beta (3 +- 3) | 0.702**

**BDNF serum | Hippocampal head | -0.11 | [-0.47, 0.29] | 70.60% | 34.00% | Beta (3 +- 3) | 0.582**

**BDNF serum | Hippocampal tail | -0.10 | [-0.46, 0.30] | 68.85% | 32.02% | Beta (3 +- 3) | 0.563**

**BDNF serum | Subiculum | -0.16 | [-0.55, 0.24] | 77.35% | 27.90% | Beta (3 +- 3) | 0.653**

**BDNF serum | CA1 | -0.14 | [-0.52, 0.26] | 72.82% | 28.68% | Beta (3 +- 3) | 0.606**

**BDNF serum | Presubiculum | -0.12 | [-0.51, 0.29] | 71.83% | 31.08% | Beta (3 +- 3) | 0.593**

**BDNF serum | Molecular layer | -0.15 | [-0.51, 0.27] | 76.05% | 29.10% | Beta (3 +- 3) | 0.642**

**BDNF serum | GC-ML-DG | 0.03 | [-0.37, 0.41] | 56.20% | 34.25% | Beta (3 +- 3) | 0.500**

**BDNF serum | CA2/3 | -0.11 | [-0.54, 0.25] | 69.60% | 33.27% | Beta (3 +- 3) | 0.569**

**BDNF serum | CA4 | -0.12 | [-0.51, 0.28] | 71.58% | 30.93% | Beta (3 +- 3) | 0.588**

**BDNF serum | Hippocampus (Vol mm3) | -0.12 | [-0.51, 0.29] | 71.38% | 30.15% | Beta (3 +- 3) | 0.622**

**BDNF serum | Duration of the disease | 0.36 | [-0.01, 0.68] | 96.10% | 8.75% | Beta (3 +- 3) | 1.97**
